# Current Approaches for Advancement in Understanding the Molecular Mechanisms of Mycotoxin Biosynthesis

**DOI:** 10.3390/ijms22157878

**Published:** 2021-07-23

**Authors:** Antonia Gallo, Giancarlo Perrone

**Affiliations:** 1 Institute of Sciences of Food Production (ISPA) National Research Council (CNR), 73100 Lecce, Italy; 2 Institute of Sciences of Food Production (ISPA) National Research Council (CNR), 70126 Bari, Italy

**Keywords:** filamentous fungi, biosynthetic gene clusters (BGCs), genomics, transcriptomics, genome editing, omics data

## Abstract

Filamentous fungi are able to synthesise a remarkable range of secondary metabolites, which play various key roles in the interaction between fungi and the rest of the biosphere, determining their ecological fitness. Many of them can have a beneficial activity to be exploited, as well as negative impact on human and animal health, as in the case of mycotoxins contaminating large quantities of food, feed, and agricultural products worldwide and posing serious health and economic risks. The elucidation of the molecular aspects of mycotoxin biosynthesis has been greatly sped up over the past decade due to the advent of next-generation sequencing technologies, which greatly reduced the cost of genome sequencing and related omic analyses. Here, we briefly highlight the recent progress in the use and integration of omic approaches for the study of mycotoxins biosynthesis. Particular attention has been paid to genomics and transcriptomic approaches for the identification and characterisation of biosynthetic gene clusters of mycotoxins and the understanding of the regulatory pathways activated in response to physiological and environmental factors leading to their production. The latest innovations in genome-editing technology have also provided a more powerful tool for the complete explanation of regulatory and biosynthesis pathways. Finally, we address the crucial issue of the interpretation of the combined omics data on the biology of the mycotoxigenic fungi. They are rapidly expanding and require the development of resources for more efficient integration, as well as the completeness and the availability of intertwined data for the research community.

## 1. Introduction

One of the main aspects that fully describes the complexity of the life of filamentous fungi is the diversity of their metabolic pathways. This implies a variety of enzymatic activities that give fungi access to a vast array of substrates and, additionally, the ability to synthesise a remarkable range of secondary metabolites, also known as natural products. In general, these bioactive compounds can play various key roles in the interaction between fungi and the rest of the biosphere, determining their ecological fitness [1]. Their production is regulated in a manner related to fungal development or in response to abiotic and biotic stressors, and the loss or overproduction of specific secondary metabolites can alter fungal development, survival, or interaction with other organisms [2]. However, beyond their biological function, many of them can have a beneficial activity to be exploited for human health, such as the immunosuppressant cyclosporin, the cholesterol-reducing lovastatin, the antibiotic penicillin, as well as negative impact on human and animal health, as in the case of mycotoxins. 

Among the most important and dangerous mycotoxins, there are aflatoxins, ochratoxins, trichothecenes, fumonisins, zearalenone, and patulin; all of them are regulated for their presence in food and feed by national and European legislation. They are produced by a wide range of fungal species and are able to contaminate large quantities of food, feed, and agricultural products worldwide, posing serious health and economic risks, by themselves or through synergistic interactions with each other [2,3]. Mycotoxins could have various functions, such as protecting the fungus from the attack of other fungivore organisms and from unfavourable environmental conditions, delimiting areas to exclude other competing mycelial networks, regulating the molecule signalling for intra- and interspecies microbial communication, contributing to the pathogenicity, aggressiveness and/or virulence of fungi [4,5]. The secondary metabolites contribute principally to the ecological fitness of fungi and therefore the molecular mechanisms underlying their production and regulation are fundamental to understand fungal evolution [2,6]. In general, the biosynthesis genes for secondary metabolites/mycotoxins are located adjacent to each other in the genome in biosynthetic gene clusters (BGCs). A single BGC can extend over tens of kilobases and typically contains most, if not all, genes involved in the production of a given secondary metabolite, although there are exceptions for which more than one cluster located on different chromosomes is required for the biosynthesis of a single compound [2,3,7]. In most BGCs, one or more key biosynthesis genes are generally present encoding the enzymes (such as polyketide synthases, terpene synthases and/or cyclases, nonribosomal synthetases, and isocyanide synthases), which define the structural backbone and the chemical class of the compounds. Other genes in the clusters encode the so-called tailoring enzymes (for example, methyltransferases, p450 monooxygenases, hydroxylases, and epimerases), which further modify the carbon molecular structure of the metabolite. In addition, genes may be present that encode proteins putatively involved in the transportation or the mitigation of the toxic property, or in the protection from the metabolite itself. Some genes located in several secondary metabolite BGCs, in the proximity of already described genes, remain unknown and need further characterisation to completely elucidate their role and establish the biosynthesis pathway. Clustering of biosynthesis genes seems to facilitate coordinated regulation of gene transcription [2,7]. Generally, a specific transcription factor gene is present in the cluster, most commonly encoding a C6-zinc cluster protein that recognises palindromic motifs in the other gene promoters. Occasionally, more than one specific regulator occurs in the BGC. In other cases, a cluster-specific transcription factor was found to regulate genes belonging to different BGCs [2]. However, the biosynthesis of fungal secondary metabolites is controlled by an even more complex regulatory network, which involves connected subnetworks. In addition to pathway-specific regulators, broad domain transcription factors contribute to both positive and negative regulation of different BGCs in all the toxigenic fungal species, responding to various biotic and abiotic stimuli. The heterotrimeric Velvet complex has been the most studied transcriptional complex that is involved in the regulation of the global secondary metabolism across every fungal genus [3,7,8]. In addition, the emerging role of epigenetic mechanisms as key regulators of fungal secondary metabolism has been recently reported. On this basis, mutation or inhibition of chromatin signalling has been widely adopted in the last 10 years to induce the expression of cryptic or silent metabolic clusters, or to discover new compounds [9,10]. 

The elucidation of the molecular aspects of mycotoxin biosynthesis has been greatly accelerated over the past decade due to the advent of next-generation sequencing (NGS) technologies, which greatly reduced the cost of genome sequencing and related omic analyses. The massive number of genomic, transcriptomic, and proteomic data associated with metabolomic data provides both evidence of new unknown compounds and clarification on the mechanism of regulation of known fungal metabolites, and in particular, mycotoxins [11,12]. In fact, the potential of combined omic approaches recently has contributed greatly to the understanding of pathogenic fungal–host crosstalk, as well as improved the comprehension of fungal ecology and production of mycotoxins in pre- and postharvest [13]. They provided new insight into toxigenic fungi genetic constitution and about mycotoxin biosynthesis by fungi in response to various ecological factors [12,14]. Furthermore, in the case of endophytic fungi such as *Fusarium* and *Alternaria*, the omic analyses applied to both the fungus and the host plant could shed new light on ecological and molecular aspects of the symbiotic relationship and the consequent mycotoxin production.

**Figure 1:**
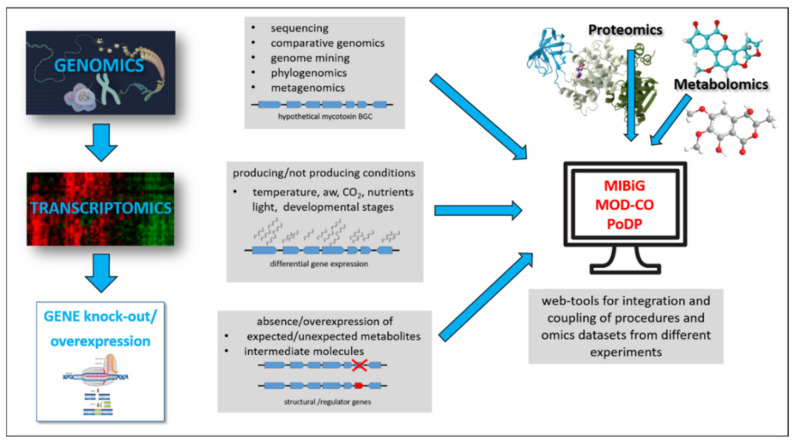
Schematic representation of experimental approaches used to identify and characterise a mycotoxin biosynthesis gene cluster (BGC).

In this context, this review briefly highlights recent progress in the use and integration of omic techniques with particular attention to genomics and transcriptomics, for the study of mycotoxins biosynthesis pathways and regulations. The genetic manipulation method, which now makes use of advanced genome-editing technologies, is also crucial for explaining the molecular mechanisms of biosynthesis by allowing the identification of key genes and their role in the pathways. Another important aspect considered here is the issue of the right management of omic data, which has become urgent as a result of the exponential increase of omic studies and repositories. In Figure 1, the workflow for the study of mycotoxin biosynthetic clusters is schematised. 

## 2. Genomics

**Figure 2:**
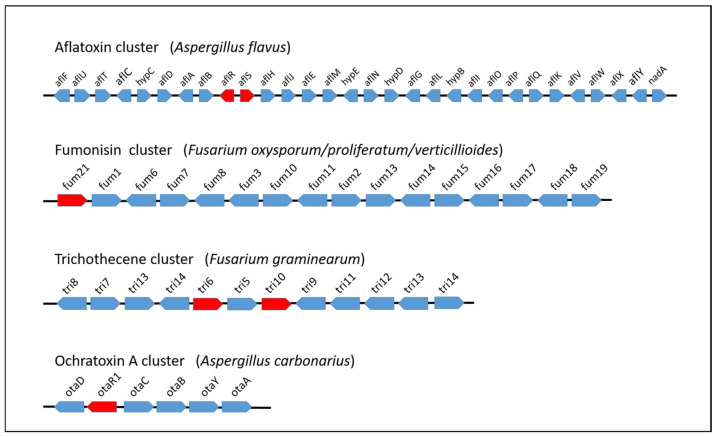
Schematic representation of mycotoxin BGCs. Lengths of genes and intergenic regions are not drawn to scale, and transcription factors are depicted in red.

In recent years, the advanced development of NGS technologies has enabled the sequencing of a large number of filamentous fungus genomes, with an exponential increase in genomic data that will continue in the near future. Most of the fungal genome data are available on the NCBI database and on several sites dedicated to fungal research, for example, Fungi DB (https://fungidb.org/fungidb, accessed 25 June 2021) and AspGD (http://www.aspgd.org/, accessed 25 June 2021), the latter of which is specific to *Aspergillus* species. Worthy of note is the recent project “1000 Fungal Genomes” at the Joint Genome Institute (JGI) for the sequencing of multiple fungal genomes; MycoCosm is the related portal (http://jgi.doe.gov/fungi, accessed 25 June 2021) at which integrated resources allow researchers to easily access and analyse a large number of fungal genomic data [15]. With regard to the fungal genera that include the most relevant mycotoxigenic species, a great number of *Aspergillus* species genomes (about 200) have been sequenced, compared to the few tens belonging to *Fusarium* and *Penicillium* genera. In some cases, the genomes of several strains belonging to the same species have been sequenced with the aim of highlighting and analysing differences due to distinct sources (food, animal, environmental, etc.) and geographic origins, as expected in a pangenomic study. Availability of genomic sequences is of great help for the identification of secondary metabolism BGCs. Filamentous fungi are producers of a diverse array of secondary metabolites, which may have highly valuable bioactive effects and potential biotechnological interest. However, most of them may not be expressed under standard experimental conditions and metabolic phenotyping does not allow their discovery [2]. In these cases, one of the most used and useful strategies is the in silico genome mining, that is the computational search in the entire genome of biosynthetic genes with particular concern for cryptic or silent gene clusters [2,16,17]. A comprehensive overview of tools and techniques for mining the fungal secondary metabolome is given in the work of Keller et al. [2]. The identification of BGCs is based on the presence of genes encoding the key synthases or synthetases characterising the structural backbone of secondary metabolite molecules (polyketide synthases, terpene synthases, and/or cyclases, nonribosomal synthetases, and isocyanide synthases). The analysis of the genomic sequence next to these key enzymes generally leads to the identification and characterisation of tailoring genes, which contribute to modify and define the final molecular structure. Among the clustered genes, specific transcription factors are generally present that positively regulate the other genes of the cluster. A schematic representation of the gene clusters involved in the biosynthesis of some of the most important mycotoxins is shown in Figure 2. In the case of the biosynthetic cluster of ochratoxin A (OTA), the exploring of a genomic region adjacent to the previously identified *pks* and *nrps* genes enabled progress in the description of the OTA biosynthesis pathway in producing species [18,19,20]. Recent advancement in the elucidation of molecular aspects of OTA biosynthesis has resulted from the comparative analysis of genomes of 21 ochratoxigenic species. This study evidenced the presence in the OTA cluster of a previously undescribed gene with a putative role in the polyketide cyclisation reaction during the initial steps of the biosynthesis pathway [21]. The comparative analyses of sequencing data among different fungal species belonging to the same genus, section, or showing some phenotypic traits in common, is crucial to find shared BGCs. In addition, these studies allow the assessment of the productive ability of a strain or species, depending on the presence or absence of the cluster or deletion of some genes whose function is essential for biosynthesis [22]. However, the peculiar architecture of some fungal BGCs split in multiple loci may not allow the identification of these biosynthetic pathways, as well as their actual functionality, through the in silico prediction based on collinearity [16]. An additional advantage provided by the comparative genomic analyses is the possibility to easily discriminate atoxigenic from toxigenic strains in a producing species. In this respect, the determination of the absence of biosynthesis genes and the presence of several genes required for high infectivity are prerequisite important for successful biocontrol agents, as in the management practice for reducing aflatoxin contamination [23,24].

### Phylogenomics

Comparative phylogenomics pairs genomics and phylogenetics by relying on advances in NGS technologies and bioinformatics pipelines that have led to remarkable revision and progress in fungal systematics and taxonomy [25,26]. Evolution mechanisms have played a major role in the biochemical complexity of fungi in relation to the production of secondary metabolites and then mycotoxins. Rearrangements in the physical organisation of the fungal genome due to duplication, combination, and modification of existing pathways, loss or gain of new genes, and therefore, acquisition or removal of enzyme activities, has led to innovations in fungal secondary metabolism as well as in the regulatory networks [5]. In addition, the acquisition of novel genes through horizontal gene transfer (HGT), often from distantly related genomes, is a relatively frequent process that has facilitated increasing and diversification of toxigenic potentiality in fungi, favoured by clustering of biosynthetic genes. Horizontal gene transfer or convergent evolution events are likely responsible for irregular taxonomic distributions of some widespread gene clusters, as highlighted by phylogenomic analyses. Hence, these studies help in identifying differences in cluster organisation and evolutionary processes responsible for structural diversification origin, along with the prediction of new potential mycotoxigenic fungi [3,5,9,27]. In a recent work on the evolution of the trichothecene cluster, Proctor et al. [28] conducted combined genomic and phylogenetic analyses, as well as gene function and analytical chemistry studies, on strains from nine fungal genera. They found the number of genes per cluster varying among genera and, in some cases, among species of the same genus, and the occurring of trichothecene genes at one to as many as five distinct loci. The hypothesis of an ancestral trichothecene biosynthetic pathway is suggested from which diverse pathways originated as a consequence of gain, loss, and functional changes of biosynthetic genes, resulting in a wide structural diversity which characterises the trichothecene molecule class [29]. A phylogenetic analysis of *Aspergillus* species belonging to *Circumdati* section was carried out by Gil Serna et al. [30] on the basis of the OTA biosynthesis cluster. They reported that the genomes of some species of recent description or rarely reported as producers contain a potentially functional biosynthetic cluster and might be able to synthesise OTA. On the contrary, some other species show a truncated version of the cluster lacking many of the biosynthetic genes and therefore incapable of production, such as *A. ochraceus* which has long been considered a major OTA producer, but it is likely that the producing strains so far isolated as *A. ochraceus* have been misidentified. It is clear that studies of this type are useful for establishing the mycotoxigenic capacities of newly isolated or described or reclassified species. In addition, some of the global regulators that control the fungal secondary metabolism, and therefore the production of mycotoxins, seem to be evolutionarily conserved across large phylogenetic distances, and their taxonomic distribution can provide some valuable details on the complexity and diversity of their functions [7,31].

## 3. Transcriptomics

Transcriptomics represents the link between genome and metabolome. Transcriptional profiling is important to understand the genes that are transcriptionally coregulated during secondary metabolites production to define both structural and regulatory genes involved. The production of metabolites/mycotoxins changes under different environmental conditions, and the impact of climate change factors can be estimated by taking into account the chemical, biochemical, physiological, and molecular aspects as a whole. In this regard, the transcriptional studies allow the estimation of the impact of climate change factors and the identification of the molecular pathways activated in response to diverse environmental cues, such as nutrient availability, light, pH, and temperature. Other conditions, concerning internal factors such as sexual cycle and developmental stage, or interaction with other microorganisms, plants, and animals, are known to influence greatly the production of mycotoxins [2,7]. The most recent and reliable approaches for transcriptomic studies are high-throughput microarray, whole transcriptome (RNA-seq) shotgun, and reverse transcription–quantitative PCR (RT–qPCR) analyses, with this latter particularly important to confirm most of the findings obtained from previous methods. With particular reference to RNA-seq analyses, more and more recently concern the assessment of the effects of changing environmental parameters (such as temperature, water activity, and CO_2_) on the regulation of the mycotoxin biosynthesis process in order to better estimate the mycotoxigenic risk associated with climate change factors. Most of these analyses regarded the biosynthesis of aflatoxin, whose molecular and regulatory mechanisms have so far been widely investigated, and stress factors have been taken into consideration acting alone or in combination [32,33,34]. The ability of RNA-seq to provide a picture of the whole transcriptome under different conditions allows the identification of activated pathways and genes that are directly involved or acting in the signalling system. There are different conditions that can be studied in addition to environmental, nutritional, or cultural parameters favouring or not mycotoxin production [35,36], such as the effect of treatment of several compounds. In transcriptomic analyses on *A. flavus*, treatments with resveratrol [37], gallic acid [38], and ethanol [39] were found to reduce aflatoxin production by enhancing the activity of antioxidant enzymes, confirming that mycotoxin biosynthesis is related to oxidative stress and functions as a secondary defence system from excessive reactive oxygen species. The RNA sequencing of deletion mutants of mycotoxigenic fungi enabled the elucidation of some regulatory mechanisms underlying mycotoxin biosynthesis. Recent works have shown that transcription factors involved in fungal conidial development and sclerotial production have a role in the pathogenesis/virulence and mycotoxin biosynthesis [40,41]. In addition, studies on deletion mutants may support the role of global transcription factors involved in signal transduction, leading to the production of mycotoxins as well as the possible activity of cluster-specific transcription factors in influencing the expression of genes in a different secondary metabolic pathway [42,43]. In a recent work on the transcriptome of *A. flavus* lacking in the homeobox domain transcription factor *hbx1,* Cary et al. [44] described *hbx1* as a global regulator that controls several thousand genes and can influence the expression of an elevated number of transcription factors and developmental regulators, together with large numbers of secondary metabolite gene clusters including aflatoxin. Among the global regulators of secondary metabolism in fungi, by far the most studied was the heterotrimeric Velvet complex, consisting of LaeA, VeA (or Vel1), and VelB (or Vel2) proteins, which was found to regulate sexual development and secondary metabolism in several mycotoxigenic fungi [2]. In particular, the main studies confirming the role of this complex were conducted on fungal strains in which *laeA* gene was deleted or inactivated, and gene expression analyses of specific mycotoxin biosynthesis genes were performed. A comparative transcriptomic study between *laeA* deletion and overexpressing mutants in *A. niger* showed that these types of analysis on global regulators allow the identification of several differentiated gene clusters and transcription factors not yet characterised [45]. LaeA protein in Velvet complex has also been suggested to function as an epigenetic regulator for its methyltransferase activity, likely linked to changes in chromatin structure [31]. Epigenetics mechanisms, such as chromatin modification and remodelling, are in fact an important aspect in the regulation of mycotoxin biosynthesis considering the characteristic structure of clusters that imply a coregulated expression of biosynthesis genes [2]. Changes in chromatin structure, particularly the switch from hetero- to euchromatin, are due to enzymes responsible for reversible posttranslational modifications of histones (among which acetylation, deacetylation, methylation, and demethylation). Relatively recent transcriptomic studies highlighted the role of these epigenetic factors in the biosynthesis of mycotoxins. The monitoring of the expression of some genes involved in the biosynthesis of fumonisin in *F. verticillioides,* in the presence of a histone deacetylase inhibitor, indicated a clear and differential role for chromatin remodelling in the regulation of FUM genes [46]. More recently, the gene *rmtA* encoding a methyltransferase in *A. flavus* was shown to regulate aflatoxin biosynthesis and fungal development, and in a subsequent RNA sequencing work, its influence on the whole transcriptome of *A. flavus* was demonstrated, identifying *rmtA*-dependent genes, including numerous transcription factors [47].

## 4. Gene Manipulation

Deletion of biosynthetic cluster genes, coupled with metabolic analysis to detect the absence of the expected metabolite and/or the accumulation of intermediate molecules, has been the most successful strategy to clarify their function and to determine the biosynthesis pathway of the most important mycotoxins [48,49,50]. Similarly, targeted deletion or overexpression of transcriptional regulators, whether they are cluster specific or globally acting, provides a precise tool for studying the regulatory mechanisms behind the production of mycotoxin. Several techniques of gene manipulation have been developed and used in filamentous fungi for this purpose, all of which have allowed a certain degree of knowledge on the biosynthesis of mycotoxins to be achieved. Nevertheless, most of these traditional methods are laborious, time consuming, and sometimes with low efficiency in gene targeting. It is, therefore, noteworthy, to focus on the recent innovation brought in this field by “Clustered Regularly Interspaced Short Palindromic Repeats/Cas9” (CRISPR/Cas9) technology. Generally, this editing method is characterised by high specificity and efficiency, versatility, and ease of operation, and its application represents a powerful tool for further research in filamentous fungi [51,52]. Schematically, the main components of this system, sgRNA and the Cas9 enzyme, enable a target double-strand DNA sequence to be cut in order that subsequently the NHEJ (nonhomologous end-joining) dominant self-repair mechanism causes a random loss, insertion, or replacement at the breakage point, resulting in gene mutation. Instead, when the HR (homologous recombination) self-repair pathway prevails, accurate editing of the target gene occurs, such as the introduction of specific point mutations or insertion of the desired sequence or the exact replacement of the target sequence if an exogenous donor DNA fragment is provided. In the last years, several variants of this technique have been developed for application in filamentous fungi. Most of them concerned the optimisation of certain steps of procedure such as the expression of enzyme Cas9, the transcription of the sgRNA, the delivery of the expression cassettes in fungal cells, the off-target integrations mediated by NHEJ, and the increase of efficiency of HR mechanism. An extensive description of the latest developments in the application of CRISPR/Cas9-mediated genome editing in filamentous fungi has been reported in recent reviews [52,53,54]. There are not many studies available at the moment regarding the use of this technique for the investigation of the molecular mechanisms of mycotoxin biosynthesis. A recent work by Ferrara et al. [55] confirmed the role of the polyketide synthase *fum1* gene in fumonisin biosynthesis in *F. proliferatum* by deleting this gene through a novel CRISPR/Cas9-based genome-editing method that allows direct delivery of preassembled Cas9 ribonucleoproteins into fungal protoplasts. One of the most interesting aspects of using the CRISPR/Cas9-based technique is the possibility of simultaneous modification of multiple genes. Currently, different methods of multigene targeting with a single transformation are available for filamentous fungi [56,57,58,59,60]. The possibility to obtain mutants in which multiple genes are deleted or inactivated appears as a valuable resource for the study of the clustered genes responsible for mycotoxin biosynthesis. Overall, the use of CRISPR/Cas9 promises easier experimental verification of BGCs under investigation and expedited construction of suitable expression systems for biotechnological processes involving mycotoxigenic fungi.

## 5. Managing of Data

The rapid expansion of data on the biology of the mycotoxigenic fungi poses the problem of their integration to better interpret and exploit them. The crossmatch of all the omic datasets in the “genomics–transcriptomics–proteomics–metabolomics” chain may result in an integrated approach that is essential to elucidate the complex system of mycotoxin production and understand the interaction between primary and secondary metabolism in fungi. In this respect, the research community is generating an increasing amount of omic data, which have been deposited in different dedicated repositories, and in the meantime, tools are rapidly being developed for mining these data [61]. In recent years, the improvement of specialised computational tools has led to the development of platforms that use genomic information to identify known BGCs in never-considered species and discover new secondary metabolites, with platforms such as the “antibiotics & Secondary Metabolite Analysis Shell” (antiSMASH) [62] and “PRediction Informatics for Secondary Metabolomes” (PRISM) [63]. Public databases such as the implemented version of the “Integrated Microbial Genomes Atlas of Biosynthetic gene Clusters” (IMG-ABC) (https://img.jgi.doe.gov/abc-public, accessed 25 June 2021) [64], antiSMASH-DB (https://antismash-db.secondarymetabolites.org/, accessed 25 June 2021), and MIBiG (https://mibig.secondarymetabolites.org/, accessed 25 June 2021) [65] have a crucial role in the analysis of BGCs, as they allow comparing the sequences of newly sequenced BGCs against those previously predicted and experimentally verified [66,67]. In this regard, it is noteworthy to highlight that computational analysis of BGCs has revolutionised natural product discovery by enabling the rapid investigation of secondary metabolic potential within microbial genome sequences. More recently, Kautsar et al. [68] have developed the BiG-FAM database, which groups homologous BGCs into gene cluster families (GCFs) facilitating the definition of their architectural and taxonomic diversity and providing insights into the novelty of putative BGCs. BiG-FAM collects data from more than 200,000 publicly available microbial genomes and metagenome-assembled genomes (MAGs), clustering more than 1.2 million BGCs into about 30,000 GCFs and offering rich functionalities for the exploration of GCFs and homology searching of BGCs (https://bigfam.bioinformatics.nl, accessed 25 June 2021). Another important and challenging aspect of managing big data on BGCs is the comparison among data and datasets from different experimental approaches as well as different data processing platforms and workstations, which may provide different information. Essential structural details and early data processing of analysis results help in obtaining a correct interpretation of the complex information provided in primary datasets [69]. This underlines the need to establish and record these steps and corresponding data pipelines from the acquisition of samples in the field, and to document identifiers at the start of data generation [70]. Interestingly, Meta-Omics Data and Collection Objects (MOD-CO), developed recently by Rambold et al. [71], has been set up as a new schema for meta-omics research, with a hierarchical organisation of the concepts describing collection samples, as well as products and data objects generated during operational workflows. Therefore, it is crucial to have available online resources for storing and linking paired omic datasets, such as genomic coupled to metabolomic data, from the same species, organism, or sample that would improve and facilitate the research and annotation studies on secondary metabolites, in particular mycotoxins, and their relevant BGCs. Recently, Schorn et al. [72] have developed the “Paired Omics Data Platform” (PoDP) to streamline access to paired omic datasets and exploit validated links between BGCs and metabolites (https://pairedomicsdata.bioinformatics.nl/, accessed 25 June 2021). The growth of multi-omics experiments has promoted the development of metadata databases, such as BioProject and BioSample [69] and, more recently, Biostudies [73], which provide information about research projects and serve as central portals of data submitted across multiple archives. In conclusion, it is interesting to cite a recent editorial by Rajesh et al. [74] on the importance of improving the completeness of public metadata associated with omic studies by researchers. In fact, the need to establish a standard for reporting metadata is emerging to ensure full and complete sharing of metadata with the broad scientific community.

## 6. Future Perspective

Other new genotype technologies and approaches are under development to explore aspects of the mechanisms of mycotoxin production. Among these, the study of microbial communities as a whole (meta-omics) may offer a more comprehensive strategy to understand the function of mycotoxins, and how their production is regulated during the interaction among both fungus–fungus and fungus–bacteria microorganisms. In this regard, the meta-omic strategy has been reviewed in detail recently [61], highlighting the key role of secondary metabolites as mediators in the microbiome interactions. The integrated exploration of large genomic and metabolomic datasets provides valuable support for the discovery of metabolites and their BGCs, as well as for the elucidation of natural product structures, the identification of their biological activities, and ecological functions. This procedure is also appropriate for the identification and analysis of genes and molecular mechanisms involved in the regulation of multiple mycotoxins biosynthesis. In particular, multi-omic data from ecological experiments on toxigenic fungi could elucidate the molecular basis of fungal interaction mechanisms regulating secondary metabolism. In fact, the mycotoxin biosynthesis pathways may be affected by levels and regulation of other mycotoxins through cross-pathway control among different BGCs within the same fungal strain or in a fungus–fungus interaction. In particular, proteome and metabolome fingerprints offer the opportunity to identify the molecular factors regulating the interaction process among fungi and host/substrate. Combined omic approaches can provide the knowledge necessary to develop more appropriate control strategies and to accelerate natural product discovery for the preserving of food safety from new fungal mycotoxin risks and for the exploitation of potential beneficial fungal secondary metabolites. Furthermore, a deeper investigation of molecular and biochemical mechanisms regulating mycotoxin production in fungi is necessary for a better understanding of the mechanism of adaptation of fungi to environmental stress conditions (resilience) and the extent of mycotoxicological hazard due to the climate change process. The relatively overall small number of genome sequences in fungi with respect to bacteria and the often low predictive value for fungal BGCs make genome mining and BGC discovery challenging in toxigenic fungi for the next future. We expect that some of these problems will be overcome with the availability of more functional genomics studies in fungi, such as from the Mycocosm project at JGI and the increasing number of biochemical studies which will provide better training data sets for the improvement of platforms for analysis of omic data.
